# Supplementary material for: In silico voltage-sensitive dye imaging reveals the emergent dynamics of cortical populations
Source: Nat Commun. 2021 Jun 15;12:3630. doi: 10.1038/s41467-021-23901-7 (PMC8206372; doi:10.1038/s41467-021-23901-7)
Supplement: Supplementary file 1 — Supplementary Information [file 41467_2021_23901_MOESM1_ESM.pdf]

Supplementary Information

In silico voltage-sensitive dye imaging reveals the emergent dynamics of cortical populations

Taylor H. Newton[1,2*], Michael W. Reimann[1], Marwan Abdellah[1], Grigori Chevtchenko[1], Eilif B. Muller[1,3,4,5], and Henry Markram[1,6]

[1]Blue Brain Project, École polytechnique fédérale de Lausanne (EPFL), Campus Biotech, 1202 Geneva, Switzerland

[2]IT'IS Foundation for Research on Information Technologies in Society, 8004 Zurich, Switzerland

[3]Department of Neurosciences, Faculty of Medicine, University of Montreal, Montreal, QC, Canada

[4]CHU Sainte-Justine Research Center, Montreal, QC, Canada

[5]Quebec Artificial Intelligence Institute (Mila), Montreal, QC, Canada

[6]Laboratory of Neural Microcircuitry, Brain Mind Institute, EPFL, 1015 Lausanne, Switzerland

*Correspondance: taylor.h.newton@gmail.com

Supplementary Fig. 1: Pairwise V_m correlations

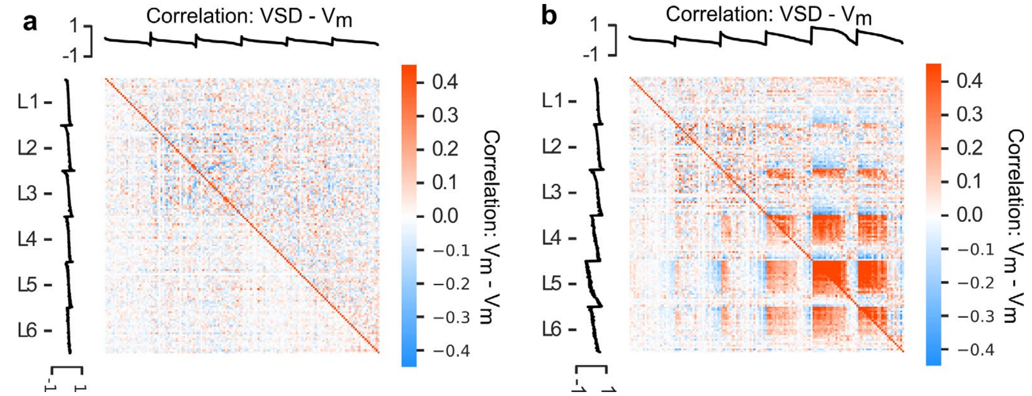

a, Pairwise membrane potential correlations between neurons (300 per layer) for spontaneous network activity. Upper triangle: correlations computed using thresholded traces (-55 mV). Lower triangle: correlations computed on raw traces including spikes. Top and left margins: V_m-VSD correlations for each cell, sorted by strength within each layer (filtered and unfiltered, respectively). **b**, Same as in **a**, but for evoked activity (single stimulus, 60 contiguous TC fibers at NMC center).

Supplementary Fig. 2: Individual NMC responses

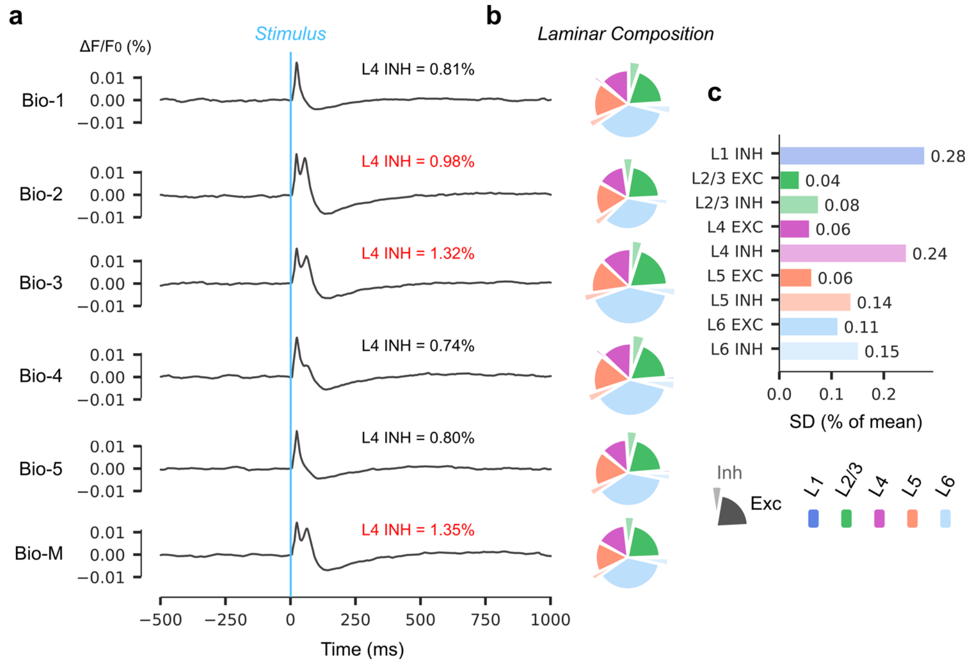

a, Spatially averaged VSD data for microcircuits based on anatomical data taken from individual rats (Bio-1-5) and a statistical average across rats (Bio-M), $[\mathrm{Ca}^{2+}]_o = 1.25\ \mathrm{mM}$. Vertical blue line indicates stimulus. Text indicates percent deviation from mean in L4 inhibitory populations (black: subcritical response; red: supercritical response). **b**, Number of cells by layer (color) and cell type (standard or exploded pie slices) for each individual microcircuit. Radius of each pie plot is proportional to the total number of neurons. **c**, Standard deviations (percent deviation from mean) for numbers of neurons by layer and cell type.

Supplementary Fig. 3: Sagittal view VSDI dynamics

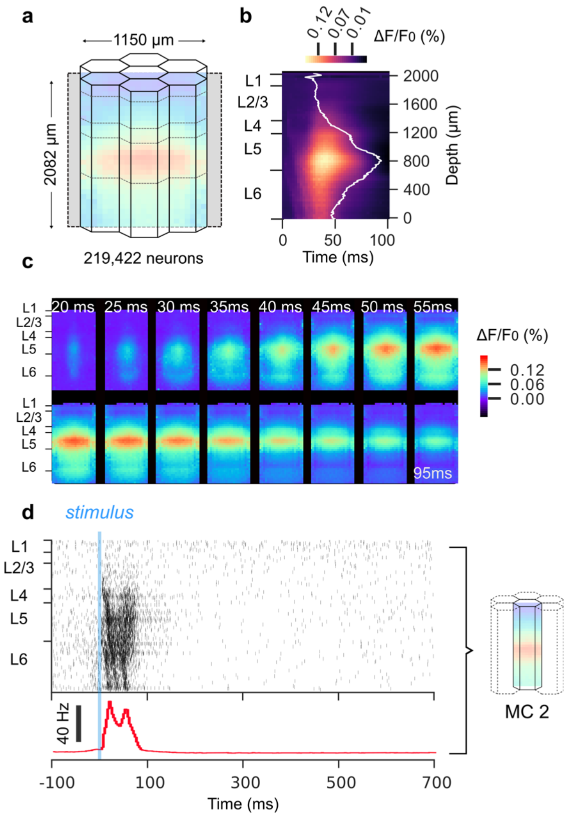

a, Mosaic configuration of NMC model (7 concentric columns), with sagittal imaging plane (indicated in gray) bisecting the volume along the y-axis (depth). **b**, Linescan of cross-sectional VSD activity: matrix of time series data for a vertical line through the center of the imaging plane. White line overlay is sum of each matrix row (i.e. the integral over time for each depth). **c**, VSDI data for a sagittal slice through the mosaic in 5 ms intervals (20-95 ms post-stimulus). **d**, Top: raster plot of 2000 randomly sampled cells in the central column (MC2). Bottom: same data as above, but in time histogram format.

Supplementary Fig. 4: Forward- and backward-propagating action potentials

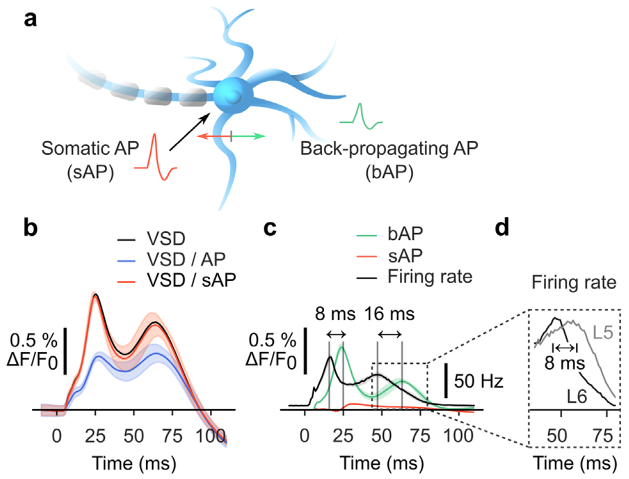

a, Schematic illustrating forward- and backward-propagating APs. Red: APs occurring at the soma/axon initial segment (sAP). Green: back-propagating APs occurring in dendritic arbors (bAP). **b**, Time domain comparison of filtered VSD signals. Black: full signal, no filtration. Blue: VSD signal computed with thresholded V_m (-55 mV), all spikes excluded. Red: VSD signal computed with thresholded V_m (-55 mV), only spikes in somatic compartments excluded. Colored error bands: standard deviation (n=10 independent trials). **c**, Comparison of VSD signal contributions by bAP (green) and sAP (red), with firing rate overlay (black). **d**, L6 and L5 mean firing rates during time window in dashed box in **c**.

Supplementary Fig. 5: Composition of reconstructed neural tissue by neurite type

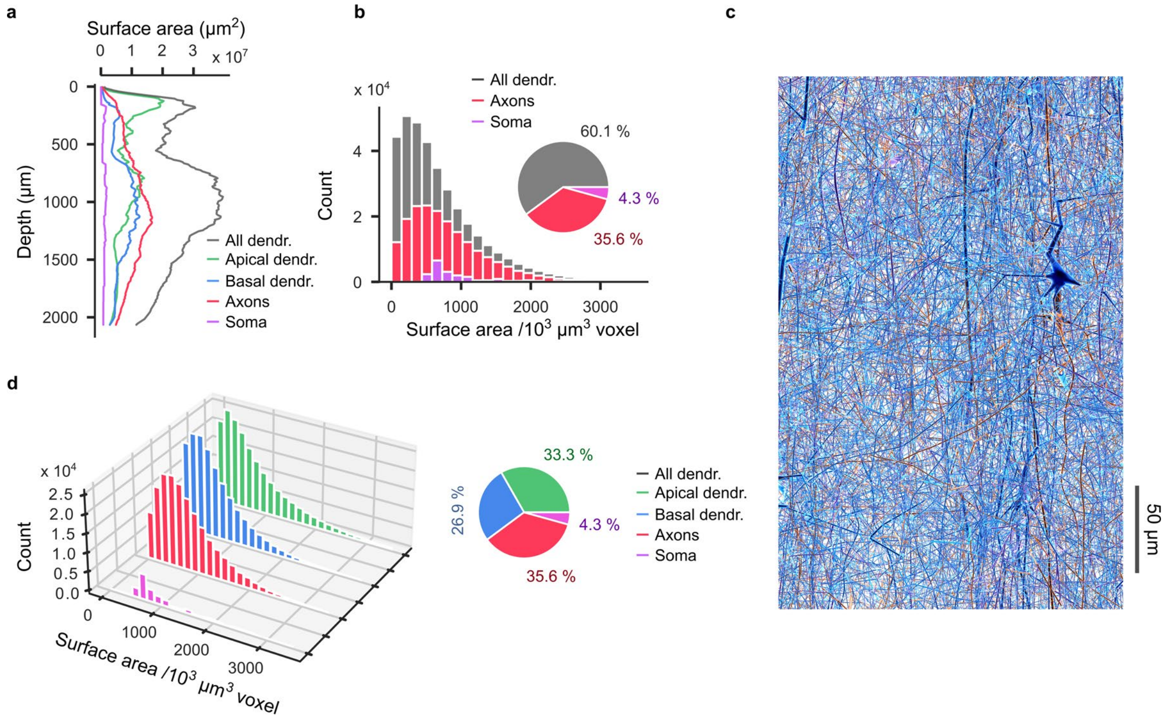

a, Distribution of surface area by depth for somatic, axonal, and dendritic compartments. Total surface area calculated by summing in 10 μm increments along the depth axis. **b**, Count histogram of surface area by compartment type for 1000 randomly sampled 10^3 $μm^3$ voxels throughout the NMC. Inset: pie chart showing percentages of surface area by compartment type over all voxels sampled. **c**, Visualization of a 200 x 300 μm section of neuropil. *Image courtesy of BBP*. **d**, Same as in **b**, but with a distinction made between apical and basal dendritic compartments.

Supplementary Fig. 6: Evoked PSTH and spike raster by neural subpopulation

a

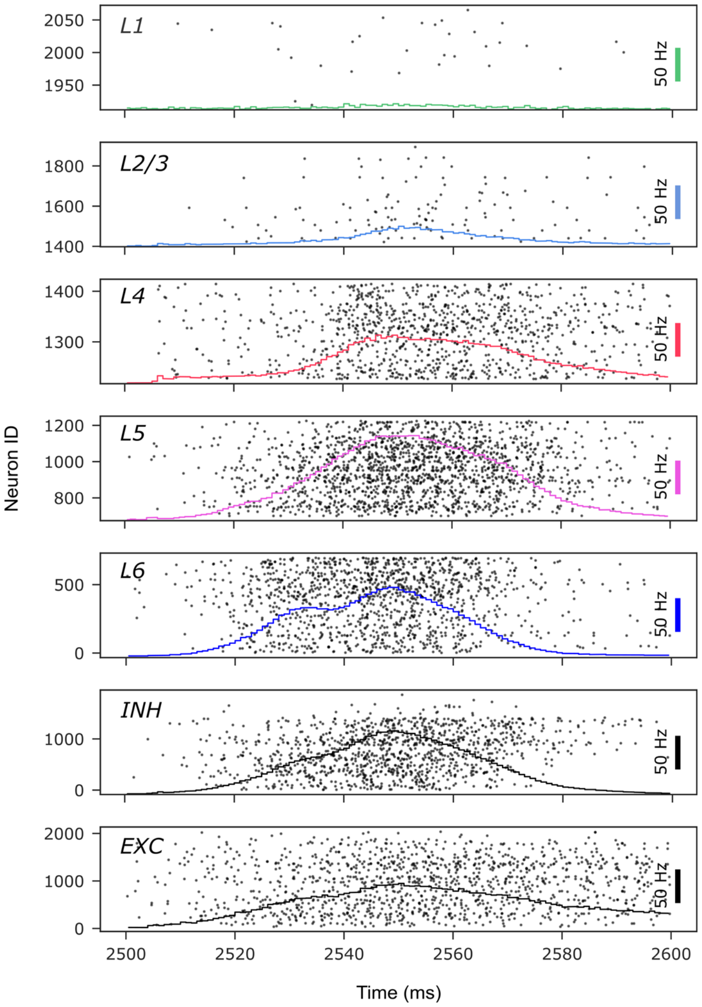

Network spiking response to VPM stimulus delivered at t=0. **a**, Spike raster for cortical layer 1 (75 randomly selected neurons), layer 2/3 (100 randomly selected neurons) and layers 4, 5 and 6 (300 randomly selected neurons) with overlaid post-stimulus time histogram (PSTH); 3 ms bins. Time of peak firing indicated with text. **b**, Same as in **a**, but for inhibitory (top panel) and excitatory neurons (bottom panel) randomly sampled from all 6 cortical layers.

Supplementary Table 1: Wavefront Propagation Velocities

Publication	***Min. speed***	***Max. speed***	***Wavefront quantification***	***Experiment protocol***	***Anesthesia***	***Brain region***	***Animal***
Petersen et al., 2003a	33 µm/ms (barrel arc)	60 µm/ms (barrel row)	Gaussian fit (cross-sectional)	whisker deflection (in vivo)	urethane or halothane	barrel cortex	rat P21-P28
Fehérvári et al., 2015	47+/-12 µm/ms	66+/-15 µm/ms	amplitude threshold (50% peak)	50 µA current injection (in vivo)	urethane	visual cortex (V1)	mouse P56-P140
Ferezou et al., 2006	27+/-7 µm/ms (urethane)	30+/-7 µm/ms (awake)	amplitude threshold (50% peak)	spontaneous (in vivo)	urethane or isoflurane or none	barrel cortex	mouse
Lippert et al., 2007	200+/-100 µm/ms	200+/-100 µm/ms	--	whisker deflection (in vivo)	isoflurane	barrel cortex	rat
Petersen et al., 2003b	<10 µm/ms	>100 µm/ms	amplitude threshold (50% peak)	spontaneous (in vivo)	urethane or ketamine/ xylazine or halothane	barrel cortex	rat/mouse P21-P35
Contreras and Llinas, 2001	181+/-44 µm/ms (L2/3)	217+/-53 µm/ms (L5/6)	--	white matter stimulation 1-5V, 100µs (in vitro)	sodium pentobarbital	visual and somatosens ory cortex	guinea pig
Chavane et al., 2011	90 µm/ms	90 µm/ms	2D Gaussian fit	sinusoidal luminance gratings (in vivo)	althesin (3 mg/kg/h) and pancuronium bromide (0.2 mg/kg/h)	visual cortex	cat
Civillico and Contreras, 2005	30 µm/ms (single whisker)	196 µm/ms (multiple whiskers)	amplitude threshold (2x SD of baseline per pixel)	whisker deflection (in vivo)	ketamine-xylazine (100 mg/kg i.p., 20 mg/kg i.p. respectively)	barrel cortex	mouse
**NMC**	**~-10 µm/ms**	**~20 µm/ms**	**2D Gaussian fit**	**whisker deflection (in silico)**	**--**	**somato-sensory cortex**	**rat**

Supplementary Table 2: VSDI Calculation Parameters

dim, res	100 μm, 100	Dimensions and pixel resolution of detector.
μ_{eff}	1.5 mm^{-1}	Effective modified Beer-Lambert law extinction coefficient.
G_0	250	Autofluorescence and noise calibration term.
V_m^0	-65 mV	Mean resting membrane potential.
f_s	2000 Hz	Sampling frequency for VSD measurements.
t_{start}, t_{end}	950 ms, 1000 ms	Start and stop times for calculation of VSDI baseline fluorescence.

Supplementary Algorithm 1: 2D Gaussian surface fit

input : A surface arr of size $n \times m$
output : A 2D Gaussian fit

`/* compute 2D surface */`
Function `2DGauss`(h, x, y, σ_x, σ_y):
    return $h \cdot \exp - \frac{1}{2}\left[\left(\frac{x}{\sigma_x}\right)^2 + \left(\frac{y}{\sigma_y}\right)^2\right]$

`/* compute first-order moments of arr */`
Function `Moments`(arr):
    total ← sum of entries in arr
    $x_0 \leftarrow \frac{1}{\text{total}} \sum$ ({row of arr} × {row index}) `// 1st moment in x`
    $y_0 \leftarrow \frac{1}{\text{total}} \sum$ ({col of arr} × {col index}) `// 1st moment in y`
    r ← row of arr at index int(x)
    c ← column of arr at index int(y)
    $\sigma_x \leftarrow \sqrt{\sum |(r - \text{index of } r) \times r|^2 / \sum r}$
    $\sigma_y \leftarrow \sqrt{\sum |(c - \text{index of } c) \times c|^2 / \sum c}$
    h ← max of arr
    return x_0, y_0, h, σ_x, σ_y

`/* compute 2D surface */`
Function `Fitgauss`(arr):
    x_0, y_0, h, σ_x, σ_y ← `Moments`(arr)
    x ← $n \times m$ matrix of row indices
    y ← $n \times m$ matrix of column indices
    def `err_fn` (x, y, arr):
        return `2DGauss`(x-x_0, y-y_0, h, σ_x, σ_y) - arr
    return `LeastSq`(`err_fn`, x, y, arr) `// least-squares fit using scipy.optimize`

`/* main routine */`
Function `Main`(arr):
    params = `Fitgauss`(arr)
