# Supplementary material for: In silico voltage-sensitive dye imaging reveals the emergent dynamics of cortical populations
Source: Nat Commun. 2021 Jun 15;12:3630. doi: 10.1038/s41467-021-23901-7 (PMC8206372; doi:10.1038/s41467-021-23901-7)
Supplement: Supplementary file 3 — Reporting Summary [file 41467_2021_23901_MOESM3_ESM.pdf]

nature research

Corresponding author(s): Taylor H. Newton

Last updated by author(s): Apr 21, 2021

Reporting Summary

Nature Research wishes to improve the reproducibility of the work that we publish. This form provides structure for consistency and transparency in reporting. For further information on Nature Research policies, see our Editorial Policies and the Editorial Policy Checklist.

Statistics

For all statistical analyses, confirm that the following items are present in the figure legend, table legend, main text, or Methods section.

n/a	Confirmed	
☐	☒	The exact sample size (n) for each experimental group/condition, given as a discrete number and unit of measurement
☐	☒	A statement on whether measurements were taken from distinct samples or whether the same sample was measured repeatedly
☐	☒	The statistical test(s) used AND whether they are one- or two-sided<br>*Only common tests should be described solely by name; describe more complex techniques in the Methods section.*
☐	☒	A description of all covariates tested
☐	☒	A description of any assumptions or corrections, such as tests of normality and adjustment for multiple comparisons
☐	☒	A full description of the statistical parameters including central tendency (e.g. means) or other basic estimates (e.g. regression coefficient) AND variation (e.g. standard deviation) or associated estimates of uncertainty (e.g. confidence intervals)
☐	☒	For null hypothesis testing, the test statistic (e.g. F, t, r) with confidence intervals, effect sizes, degrees of freedom and P value noted<br>*Give P values as exact values whenever suitable.*
☒	☐	For Bayesian analysis, information on the choice of priors and Markov chain Monte Carlo settings
☒	☐	For hierarchical and complex designs, identification of the appropriate level for tests and full reporting of outcomes
☐	☒	Estimates of effect sizes (e.g. Cohen's d, Pearson's r), indicating how they were calculated

Our web collection on statistics for biologists contains articles on many of the points above.

Software and code

Policy information about availability of computer code

Data collection	• EMSim is a custom software package for the efficient calculation of data volumes (see Equation (2.1)) resulting from electromagnetic field-dependent biophysical signals. This software was used to compute raw VSD volumes, and is freely available at: https://doi.org/10.5281/zenodo.4725578.<br>• All code pertaining to the in silico VSDI pipeline (i.e., the post-processing of above volumes into VSD imaging data; see Equations (2.2) through (2.6)) is open source and available at: https://doi.org/10.5281/zenodo.4725548.<br>• Our extension of the Physically-based Rendering Toolkit (PBRT) (Pharr et al., 2016) for computing Monte Carlo simulations of photon interactions in highly turbid media is freely available at: https://github.com/BlueBrain/pbrt-v2.
Data analysis	• All analysis code (i.e. code used to generate figures from VSD imaging datasets, together with the datasets themselves) is freely available for download at: https://doi.org/10.5281/zenodo.4733519

For manuscripts utilizing custom algorithms or software that are central to the research but not yet described in published literature, software must be made available to editors and reviewers. We strongly encourage code deposition in a community repository (e.g. GitHub). See the Nature Research guidelines for submitting code & software for further information.

Data

Policy information about availability of data

All manuscripts must include a data availability statement. This statement should provide the following information, where applicable:

- Accession codes, unique identifiers, or web links for publicly available datasets
- A list of figures that have associated raw data
- A description of any restrictions on data availability

• The models and circuit data used for this study are freely available for download at: https://bbp.epfl.ch/nmc-portal/downloads
• The raw data (post-processed) required for reproduction of all manuscript figures (main and supplementary) is freely available for download at: https://doi.org/10.5281/zenodo.4733519
• All figure data is freely available in tabular format (Excel) at: https://doi.org/10.5281/zenodo.4733519
• The raw, pre-processed data, i.e. raw compartment voltages and VSD volumetric datasets, are available upon request. They are not able to be uploaded to a data hosting repository due to their prohibitive size (170 TB).

Field-specific reporting

Please select the one below that is the best fit for your research. If you are not sure, read the appropriate sections before making your selection.

☒ Life sciences ☐ Behavioural & social sciences ☐ Ecological, evolutionary & environmental sciences

For a reference copy of the document with all sections, see nature.com/documents/nr-reporting-summary-flat.pdf

Life sciences study design

All studies must disclose on these points even when the disclosure is negative.

Sample size	In figures 2 through 4, for all analyses reporting statistical quantities, we used N=10 trials per condition. Each trial was simulated by selecting a unique random seed to initialize stochastic circuit parameters (i.e. probability of synaptic failure, ion channel noise, spontaneous synaptic release probability, and noisy somatic depolarization; for further details see Nolte et al., Nat. Commun., 2019). The choice of N=10 represents a trade-off between statistical power and data storage requirements, as each trial occupies roughly 5 TB of hard disk space. In figure 6 (two-point discrimination), N=25 trials were conducted per stimulus location, as a greater number of trials were necessary to produce sufficient data points for a receiver operating characteristic (ROC) analysis.
Data exclusions	In figure 4 (detectibility of spiking activity in VSDI signals), a single trial was dropped from the analysis due to corruption (of unknown origin) in the simulation results file.
Replication	N/A. All experiments were performed in silico, and are therefore exactly reproducible given a suitable hardware configuration and software environment. Scripts, software requirements, and post-processed datasets for replicating figure data are freely downloadable as described in the software/data availability statements.
Randomization	N/A. The analysis presented here did not involve partitioning individuals into groups.
Blinding	N/A. All experiments and analysis were entirely programmatic/automated.

Reporting for specific materials, systems and methods

We require information from authors about some types of materials, experimental systems and methods used in many studies. Here, indicate whether each material, system or method listed is relevant to your study. If you are not sure if a list item applies to your research, read the appropriate section before selecting a response.

Materials & experimental systems

n/a	Involved in the study	
☒	☐	Antibodies
☒	☐	Eukaryotic cell lines
☒	☐	Palaeontology and archaeology
☒	☐	Animals and other organisms
☒	☐	Human research participants
☒	☐	Clinical data
☒	☐	Dual use research of concern

Methods

n/a	Involved in the study	
☒	☐	ChIP-seq
☒	☐	Flow cytometry
☒	☐	MRI-based neuroimaging
